# An Efficient Surface Map Creation and Tracking Using Smartphone Sensors and Crowdsourcing

**DOI:** 10.3390/s21216969

**Published:** 2021-10-20

**Authors:** Md. Rabiul Ali Sarker, Md Hassanuzzaman, Purnendu Biswas, Saikot Hossain Dadon, Tasmina Imam, Tanzilur Rahman

**Affiliations:** Department of Electrical & Computer Engineering, North South University, Dhaka 1229, Bangladesh; sarker.rabiul@northsouth.edu (M.R.A.S.); md.hasanuzzaman@northsouth.edu (M.H.); biswas.purnendu@northsouth.edu (P.B.); saikot.dadon@northsouth.edu (S.H.D.); tasmina.imam@northsouth.edu (T.I.)

**Keywords:** smartphone, navigation application, sensors, map, crowdsourcing

## Abstract

Like Smart Home and Smart Devices, Smart Navigation has become necessary to travel through the congestion of the structure of either building or in the wild. The advancement in smartphone technology and incorporation of many different precise sensors have made the smartphone a unique choice for developing practical navigation applications. Many have taken the initiative to address this by developing mobile-based solutions. Here, a cloud-based intelligent traveler assistant is proposed that exploits user-generated position and elevation data collected from ubiquitous smartphone devices equipped with Accelerometer, Gyroscope, Magnetometer, and GPS (Global Positioning System) sensors. The data can be collected by the pedestrians and the drivers, and are then automatically put into topological information. The platform and associated innovative application allow travelers to create a map of a route or an infrastructure with ease and to share the information for others to follow. The cloud-based solution that does not cost travelers anything allows them to create, access, and follow any maps online and offline. The proposed solution consumes little battery power and can be used with lowly configured resources. The ability to create unknown, unreached, or unrecognized rural/urban road maps, building structures, and the wild map with the help of volunteer traveler-generated data and to share these data with the greater community makes the presented solution unique and valuable. The proposed crowdsourcing method of knowing the unknown would be an excellent support for travelers.

## 1. Introduction

Various sports and outdoor activities, such as survival in the wild, have become extremely popular worldwide in recent years. Tracking is of vital importance for travelers to avoid getting lost and risking their lives. Sightseers like hikers and cyclists require lots of information to guide them through any sort of environmental and road conditions. Google Maps is more famous for outdoor navigation, but it cannot navigate the places where travelers have more interest to explore, such as hill tracks and waterfalls. Explorers need a travel guide to analyze the area since there is no exact route found on Google Maps or other mapping applications.

Similarly, it is not easy to find the mega infrastructure’s desired location such as a university, shopping mall, airport, or hospital since very few provide a digital indoor map. Smartphone navigation is for the omnipresent outdoors, but there is no helpful information about the mega indoor infrastructure. For this reason, reliable indoor and outdoor mapping and localization are in great demand nowadays. Researchers are trying to find optimal localization methods and suitable, innovative user-interfaces for overcoming this problem using a smartphone.

Previously, several applications were developed to provide valuable tools to advance outdoor mapping technology. The use of the embedded system with sensors for such purposes has become progressively common. Integration of embedded systems with the Internet of Things (IoT) has also been explored for tracking [1]. IoT-enabled, sensor-based devices are changing the choice of travel mode of people [2]. Commercial products currently available in the market and used for mapping or tour guides are expensive for travelers [3]. Velasquez, N. et al. recently developed a low-cost IoT Prototype for outdoor tracking with cloud service [4] that allows users remote access and to see spatial information with the data acquired from the tracking device. Using passive Radio-Frequency Identification (RFID) technologies, Rehman, S. developed a real-time and offline location tracking system [5]. 

Due to recent technological advances, smartphones increase pervasive computing ability in different types of functions. Using the varieties of sensors available in the smartphone, researchers have developed a mapping and navigation system. This contains location information of walkers, indoor spaces, and comprehensive indoor maps and models for route planning, navigation direction, and several other applications. Inertial sensors, accelerometers, and gyroscopes, which are accessible on most smartphones, are used to position indoor localization [6,7]. Different smartphone applications have also been developed for outdoor navigation and collecting spatial information [8]. Zhao, Y et al. [9] developed a real-time bicycle record system on IoT using a smartphone and an embedded system, which required an additional sensor reed switch and microcontroller to transmit the data. Additionally, there is no other system that records indoor mapping information.

In this work, it was hypothesized that the smartphone sensors can be used to create route maps of unreached areas and surface maps of infrastructure efficiently and that the number of route maps can be increased by using crowdsourcing to make the experience of travelers handy. Towards this, a novel system was proposed, developed, and tested that can be used to create a map and collect spatial information of routes and infrastructures using smartphone sensors as a simultaneous localization and mapping (SLAM) method. Mapping is done automatically by collecting sensor information from smartphones carried by the users. The application is designed based on four smartphone sensors: Accelerometer, Gyroscope, Magnetometer, and GPS, which are available even in a low-end smartphone device. It is capable of performing surface and outdoor mapping worldwide with feedbacks from crowdsourcing. The surface maps are verified by proper authorities or owners of the designated places before sharing with the users. In the case of discovering the unmapped region, the application mainly relies on travelers who explore unreached remote and hilly areas or familiar public places. In this way, the proposed solution can better cover the less developed areas than other applications designed for similar purposes. 

The solution comes in handy as travelers can create and share the maps with few clicks. Furthermore, it is affordable for all travelers. It does not require an additional battery source and does not necessitate users to remain online all the time. The proposed solution is much simpler than the actual state of the art and is accessible to a larger user pool. 

## 2. Related Works

The present mobile technology offers new approaches to collect environmental data through mobile sensors which improve the existing navigation techniques [10]. The drive-by system is used in the City Scanner as a form of mobile sensing [11], dedicated in creating cost-effective data acquisition to monitor an urban environment using sensors installed in vehicles that roam around the city. Chang et al. [12] states the usability and importance of GIS in the current mapping system and analyzes the spatial information provided by the user device. Our proposed system incorporates GIS for data acquisition by taking background information from the user as a volunteer and, secondly, when mapping starts using the location-based services like a fused location provider in Google Play Services. The Data management is done by using a firebase Real-Time database management system, whereas the data query is done by using a NoSQL query. Vector data analysis uses both points and lines to create route paths and polygons for surface map creation. To display data in the proposed system, the Mapbox service is used. Embedded sensors are used in the BikeNet model [13], which emphasizes improving bikers’ experiences by providing users with feedback on bike performances and the environment around it with an app-based and web page-based interface using cellular data communication as well as the wireless access point. It provides a sharing option to the user to share routes to interested groups while environmental data is shared with everyone who has access to the app or webpage. To help tracking of parcel items to be more convenient for smaller transit agencies, EasyTracker developed a simple mobile-sensing solution [14] that incorporates the use of a smartphone that has an app with a built-in algorithm placed inside delivery vehicles to detect the routing path using GPS and to calculate duration requirement for delivery schedule. Researchers also studied simultaneous localization and mapping using foot-mounted inertial sensors [15] and a 2.5-d laser scanner [16]. The mobile-sensing platform with crowdsourcing can be highly advantageous to society, especially when addressing natural or human-made catastrophic situations [17]. The use of such a technique in such an emergency can be proven wise and time worthy. 

Different types of method have been used for mapping based on crowdsourcing. The first crowdsourcing-based indoor localization using fingerprinting-based approaches was proposed by Pie et al. [18]. Chang et al. [19] and Wilk et al. [20] proposed the system that utilizes the crowdsourcing Wi-Fi fingerprints to obtain the radio map. A robust crowdsourcing-based indoor localization system can automatically construct the radio map using crowdsourcing data collected by smartphones [21]. The magnetic trajectory data obtained through crowdsourcing are used to create the indoor map [22]. By crowd-sensing, Chon et al. [23] focused on characterizing the indoor spaces with photos and audio clips from the users. Five different images and audio classifiers were used to summarize the characteristics of a different place.

Most of the research works focused on developing solutions using existing map technology and are not entirely similar to the proposal. To better discuss this context, some of the currently available and popular map applications were reviewed. OpenStreetMaps (OSM), Mapillary, and Strava are currently available mapping applications with similarities with the proposed work. OSM is an open-source mapping platform that contains geographic information fully contributed by the volunteer users. It is built by a mapping community that creates and maintains data about roads, trails, Cafés, railway stations, and much more worldwide. The best thing about this app is that it can work on multiple device modes, such as websites and mobile apps. Since the volunteer contributors of OSM own, modify, and share data publicly, this app may become vulnerable to mapping vandalism. The users have to use the data at their own risk as no quality check is performed. The accuracy of OSM coverage may vary. For example, in south Asian regions, many essential streets, parks, and public areas are missing. It means some places have garnered much more attention than others. Mapillary is another popular mapping application that makes routing possible in different locations using street-level imagery and map data worldwide. Using the pictures uploaded by the users, they can create dots over any street on the map where each dot signifies one picture of the surrounding as uploaded and updated. The major drawback with Mapillary is that it requires image files from the users. Uploading the same-quality, full images from all parts of the world may not always be possible due to inadequate acquisition devices and network coverage. Strava, on the other hand, is best known for its GPS-aided cycling and running app, especially favored by athletes or fitness-conscious people. It allows users to connect to multiple devices such as smartwatches for biometric signals, for friends’ quests, and for performances. It mainly targets athletic-minded people and, therefore, many people outside this circle might find this application less interesting.

## 3. System Overview

**Figure 1:**
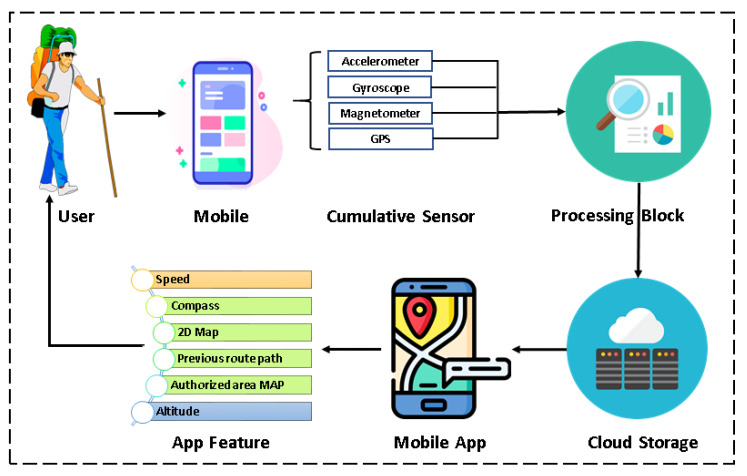
System architecture showing the detailed activity of the proposed solution.

The proposed system is shown below with the help of a graphical flowchart in Figure 1. The primary sensors needed for this app are Accelerometer, Gyroscope, Magnetometer, and GPS. The collected information from these sensors is used to produce both surface and outdoor maps using the software algorithm.

The system is dependent on both the hardware and software of the smartphone of the users. The application seeks permission from the user to use some of the sensors available in the smartphone architecture. After getting permission, it uses data from the primary sensors to create a map. The map data are updated continuously to the cloud storage for further access by the current user or anyone else having the same application installed in their smartphone. The proposed system allows accessing and sharing aggregated information of retrieved sensor data in a simple yet informative Graphical User Interface (GUI) through cloud storage. The users can see their uploaded files and files of others by accessing the cloud storage through their GUI. 

### 3.1. User

Users travelling in the wild or stuck in the urban city traffic and looking for a quick way out can use this mapping application. Users can access shared paths or share created paths themselves. After installing and providing necessary permissions, they can access features, as shown in Figure 1. Furthermore, travelers can create a landscape map or locate the boundary of infrastructure that is currently unavailable in Google Maps. Travelers are the valuable entities of this proposed crowdsourcing method.

### 3.2. Hardware

The specification requirement of the application can be divided into two categories: hardware and software. The hardware requirements are the availability of the necessary sensors and their data retrieval rate. In contrast, the software requirement is the Android OS version support for using these sensors to run smoothly. The required sensors for the application are Accelerometer, Gyroscope, Magnetometer, and GPS sensor. The existence of these sensors is imperative for the proposed application’s functionality. These are very commonly available on all low-end or high-end Android devices. The Accelerometer, Gyroscope, and Magnetometer are handled using Sensor API provided by Android OS. Each of the sensors uses a three-axis coordinate system to express the data-values.

Each axis, in meters per second squared (m/s^2^) unit, measures the acceleration applied to the device. This sensor’s data include gravitational acceleration, which needs to be filtered out to get actual acceleration. It also shows the acceleration direction of the user. This actual axis-wise acceleration can then be used for distance calculation and determining road condition, path type, and step count. 

A gyroscope [24] measures the device’s angular velocity in radians per second (rad/s) in all three axes. A particular axis’s angular velocity is the device’s angular velocity, fixing that respective axis as the axis of rotation. Gyroscope values can help to determine the direction of movement by recording the device’s axes’ displacement from their initial positions. The application gathers accelerometer and gyroscope data points using the functions called *Sensor Changed* and *Accuracy Changed*.

**Figure 2:**
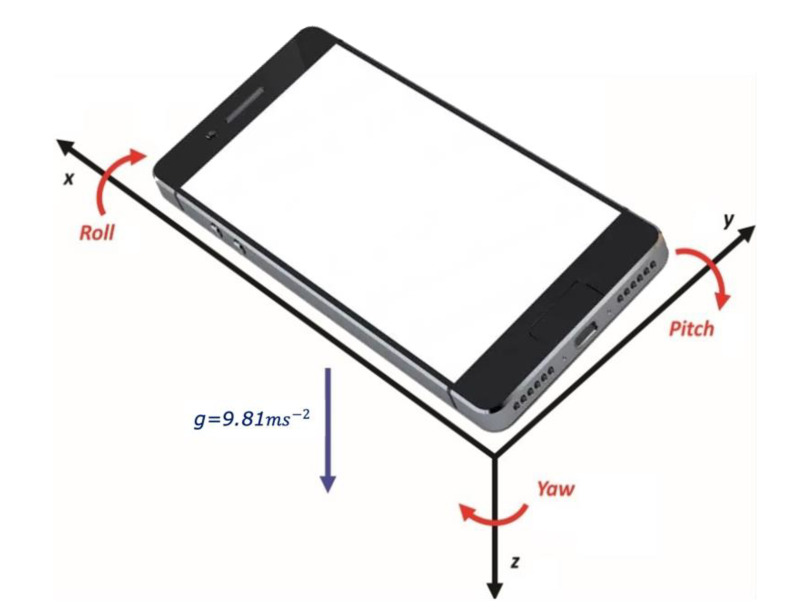
Getting Roll, Pitch, and Yaw data from the device.

The geomagnetic field sensor [25] monitors changes in the Earth’s magnetic field. This sensor provides raw field strength data in micro-Tesla (μT) for each of the three-coordinate axes. The raw data with acceleration data are used to get the rotation matrix and to calculate the orientation angles shown in Figure 2. Only three recordings, of Yaw, Pitch, and Roll, are required to develop the proposed system. Yaw measures the angular displacement of the phone’s top edge compared to the North Pole, and Pitch represents the angle between the planes parallel to the device’s screen. Ground and Roll contain the angle between perpendicular planes of both the device’s screen and the ground. 

GPS [26], Global Positioning System, is one of the vital sensors needed and used in various mapping applications, whether embedded or just an application. This sensor provides longitude, latitude, altitude, and time value as coordinates. This means the application can measure user orientation at all times, from the beginning to the end. Furthermore, using data from this sensor, user location can be verified and be kept in check constantly.

### 3.3. Software

**Figure 3:**
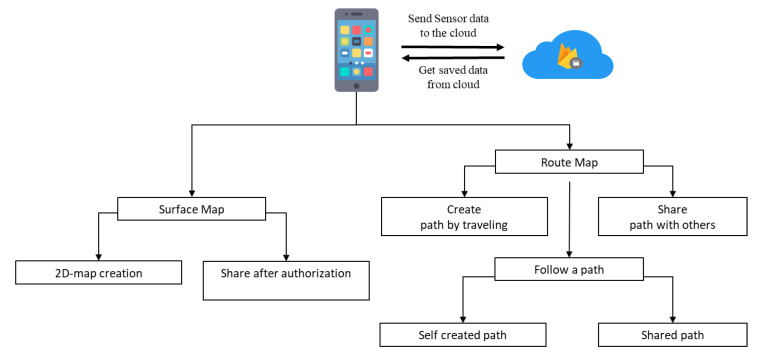
Key features of the smart travelling application.

The workflow of the software functionalities of the proposed smartphone application is provided in Figure 3. When the user wants to follow any path, the application continuously sends and receives data from the cloud. The system architecture checks the longitude and latitude values from GPS and compares them with everything available in the Cloud storage. If it matches any, it is considered a known map, and the GUI shows the map by loading the information from the cloud. If it does not match any of the existing map information, then the coordinate is considered as unknown. For unknown coordinates, the user may choose to create a map based on his/her tread on the applications. Route Map and Surface Map are the two essential features of this solution.

#### 3.3.1. Route Map

The proposed application may help users find their destination or adventure through the following options: Create a path, Share Created Path, Follow the self-created path or path shared by others. A user or authority can create the map by the system application. At first, the user needs to travel an unmapped area with a guide or other means with the system. For creating a path, the user has to press *create a route* tab before starting the journey. During the trip, the application uses primary sensor data to acquire the user’s orientation and the environmental data to create a 2D map at the end of the journey. Map creation is possible for any unmapped areas and city/urban areas. Unmapped regions are not currently available on the existing map application, such as hill tracts, rural areas, forest areas, and trails. Once the application has developed the route, users can upload it to the cloud for others to access it. When a user creates the first path, it is stored in the database and can only be accessed by the creator. When the creator shares the created path, it becomes available for other users. In this way, the application can show more path combinations than any other application without actual surveillance.

The user can follow a self-created path or paths shared by other users by following a path option. The user who created the map can access his/her creation anytime in need as it is saved in Cloud Storage. If required, they can keep it in their local storage. All saved maps in the local storage can be viewed in the application without requiring any internet connectivity. In this manner, it is easier for the users to navigate their destination without constant dependency on or paying for the internet connection. Any user willing to go out and have sanctuary in the wild or have trouble finding a specific area in the city can search for maps created and shared by other users. Accessing maps created by other users only requires wireless connectivity to download the cloud storage map file. Upon using the shared map, the user can give feedback or rate the map. This adds trust towards a shared map for others who are willing to follow. Users who are using the shared path can suggest any change in landmarks and structures around the map to keep it continuously updated so that anyone in need can have better navigation every time.

#### 3.3.2. Surface Map

The application offers users authority to create maps of infrastructure, water resources, and parks available on land for surface mapping. The application is beneficial for creating an internal map of a large institution such as a University, Industry, or Amusement Park. Surface mapping of infrastructure can be made by the respective authority using a surface tab option and shared with others through the cloud. The sensors help understand altitude, user orientation, longitude, and latitude to create a complete map. If an authority wants to edit the surface maps or needs any changes, they can do so with this application. Currently, no other applications provide the feature of creating a 2D structural maps.

**Figure 4:**
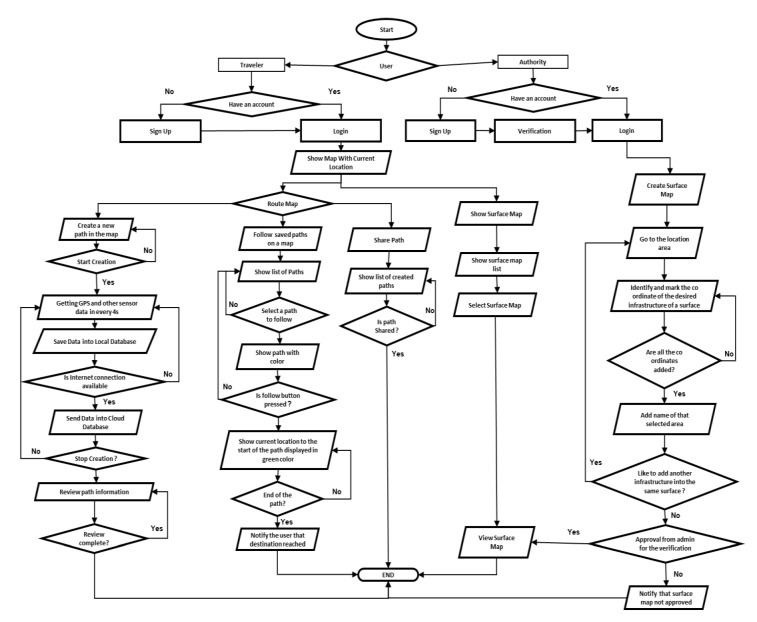
The detailed workflow of the proposed application.

If a surface map for an infrastructure, organization, or building is currently unavailable, any user can create these facilities’ maps. However, such maps are first forwarded to the authority for verification and approval. They are then released over the cloud for everyone to access. Authorities of any infrastructure can access the application through the institution page, authorize maps that have been created based on their infrastructure, and provide necessary adjustments. Users without approval from the authorities of that infrastructure will not be able to share the map. In this way, the solution ensures the reliability of the surface maps. A detailed flowchart of the proposed solution is also provided in Figure 4. It is important to note that none of the existing mapping solutions, such as Open Street Map, Mapillary, or Strava, give the surface mapping facility. It is, therefore, a unique feature of this application.

## 4. System Implementation

**Figure 5:**
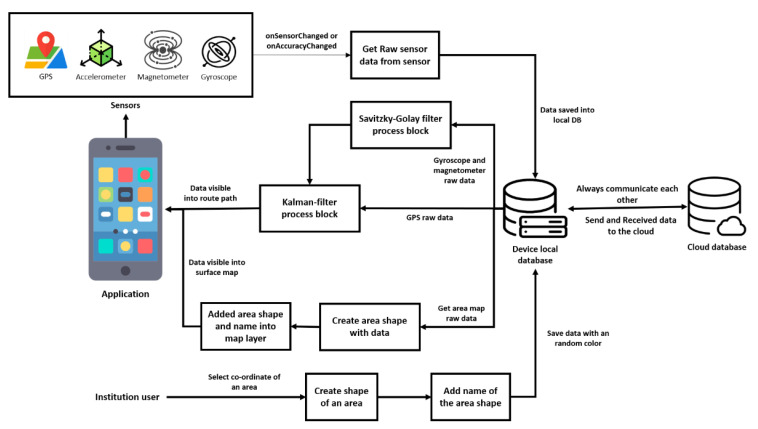
The system architecture of proposed application.

The system architecture of this application is illustrated in Figure 5. When the user starts using the app at first, raw sensor data get stored in the local database of the device. The raw data of a gyroscope and magnetometer are processed through a Savitzky–Golay filter and Kalman filter, whereas GPS raw data are processed through a Kalman filter only. These two sets of data are then available in the app to create a route path. There is always communication between a local database and cloud database and, so, the data are always updated. Therefore, the raw data of an area map from the local database help to create an area’s shape and name to get a surface map in the application layer. Similarly, Institution users do the same and save the data in the local database/device. This mechanism goes on as long as there is a change in sensor data. 

### 4.1. System Requirements

Software requirements are set based on the application’s two core components: fetching GPS location using Google Play Services and other sensor data using Android Sensors API. Sensor availability varies between different Android versions. A particular sensor may be introduced in one Android version, but its implementation in the Sensors API can arrive through a series of platform releases. The required sensors for the proposed system and their availability in different android versions are given in Table 1 [27]. The gravity sensor is a virtual sensor implemented in the Sensors API using data from other sensors.

The minimum system requirement for the users to drive all the application’s functionalities is Android Version 2.3, API level 14, as shown in Table 2. However, for fetching GPS location, the application uses the Fused Locations Provider, an API of the Google Play Services library. Google Play Services are not supported by devices older than Android 4.1. Therefore, the minimum software requirement is **Android 4.1 (API level 16)** [28]. Android 4.1 (Jelly Bean) is quite an older version, and, as such, the cumulative user percentage is as high as 99.8%.

Another requirement for the smooth and accurate performance of the application is the sensor data retrieval rate. Table 2 shows the required data retrieval rates in milliseconds for each of the sensors.

### 4.2. Location Fetch Method

**Figure 6:**
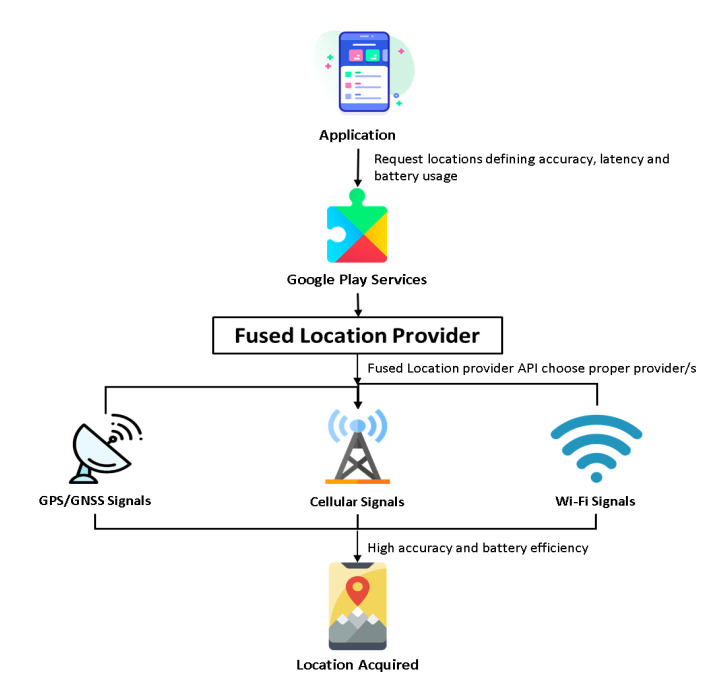
Location Provider API workflow.

Information about the user’s location, such as latitude, longitude, and altitude, can be derived from multiple sensors available on smartphones. Applications need to request the Android OS to access location information by specifying a location provider like GPS or network-based providers. However, deciding the right combination to ensure accuracy and battery efficiency is a challenging task. Fortunately, the fused location provider API [29] included in Google Play services can smartly manage the hassle to allow applications to work with accurate and battery-saving access to user location efficiently. Alternately, the area can be accessed by using the Android Location Services Framework. However, research on battery inefficiency using Android APIs [30] shows that the Location Manager API of the android framework is inadequate for efficient energy consumption. An evaluation study done by the U.S. National Institute of Standards and Technology (NIST) [31] showed that the Indoor localization accuracy of fused location provider API outperforms other popular location-fetching methods like Apple’s Core Location. The same research also reports that the fused location provider falls not far behind PerfLoc (Performance Evaluation of Smartphone Indoor Localization Applications), the winner of a competition [32,33] given the API’s limited resource requirements. The workflow of the fused location provider API is depicted in Figure 6.

### 4.3. Improving Accuracy of Sensor Data 

GPS works nicely in detecting one-time positioning values. However, its accuracy reduces while dealing with the stream of data. This accuracy is affected by several factors that include various surrounding conditions. The Kalman filter has been used in this project to ensure accurate positioning, vital in the navigation system of a moving object. The Kalman filter estimates vectors with accuracy represented by covariance matrices. The android operating system makes this estimation simple as location providers in Android give the location in longitude and latitude with accuracy specified in a single unit of meters. All the SI units involved, such as longitude, latitude, and meters, can be ignored during scaling. 

The Kalman filter is a set of mathematical formulas that provides an effective recursive solution of the least-square technique. It can provide an estimation of past, present, and also future states [34]. It is also used to remove the noise to get a precise value. In the proposed Application, the Kalman filter is used to fuse direction information, speed, and road to get the optimal current position. It is based on smartphone orientation sensor data, which is a combination of accelerometer and magnetometer. One of the magnetometer output azimuths is the angle distinction between geographical North and smartphone pointing direction. This angle is determined by magnetometer and it is affected by an electronic device like a smartphone. The gyroscope provides angular acceleration without affecting any electronic device and this value is used to compensate the effect. Before using the raw signal of the compass, it needs to be filtered as its noise is generally high [35]. For denoising the azimuth signals, which are received from mainly pedestrians, the low pass filter only is not enough for accurate data. Therefore, the raw signals are applied with a transient Savitzky–Golay filter. Then, the data are processed with the Kalman Filter with the filtered angle.

At time stamp t, θ_t_, ω_t,_ and αt are considered to be the angle, angular velocity, and angular acceleration. The system state is represented as

S_t_ = [θ_t_→ω_t_]^T^ (1)

and αt is defined as the input of the system. By following Newton’s Law, we can define the system as:

S_t_ = AS_t−1_ + Bα_t−1_ + w; (2)

where is the coefficient $A=\left[\begin{array}{cc} 1 & dt\\ 0 & 1 \end{array}\right]$, $B=\left[\begin{array}{c} dt^{2}/2\\ dt \end{array}\right]$ and w is the Gaussian noise of the system with zero mean and variance ϕ. The system observation comes from one of the orientation sensors’ outputs, the azimuth value that is denoted as O_t_. The observation function can be expressed as:

O_t_ = CS_t_ + n; (3)

where C = [1 0] and n defines the Gaussian noise of the magnetometer output with zero mean and variance, φ.

To solve this linear problem, the Kalman filter [1] is applied with the assumption of Gaussian noises. The expression contains two parts:

Predicting:

S_t|t−1_ = AS_t−1|t−1_ + Bα_t_ (4)

P_t|t−1_ = AP_t−1|t−1_A^T^ + ϕ; (5)

Here P_t|t−1_ is the estimate error. It is defined as

P_t|t−1_ = E[e^−^_t_ − e^−T^_t_] (6)

where e^−^_t_ and e^−T^_t_ are the priori and posteriori estimate errors, respectively, 

Updating:

S_t|t_ = S_t|t−1_ + K_t_(O_t_ − CS_t|t−1_) (7)

Here, the difference (O_t_ − CS_t|t−1_) in Equation (7) is called the measurement residual.

K_t_ = P_t|t−1_C^T^(CP_t|t−1_C^T^ + φ)^−1^ (8)

K_t_ = P_t|t−1_C^T^/(CP_t|t−1_C^T^ + φ)

From Equation (2), the gain K_t_ weighs the residual more heavily when variance φ of the measurement approaches zero.

P_t|t_ = (I − K_t_C)P_t|t−1_; this equation defines an a posteriori error covariance estimate.

**Figure 7:**
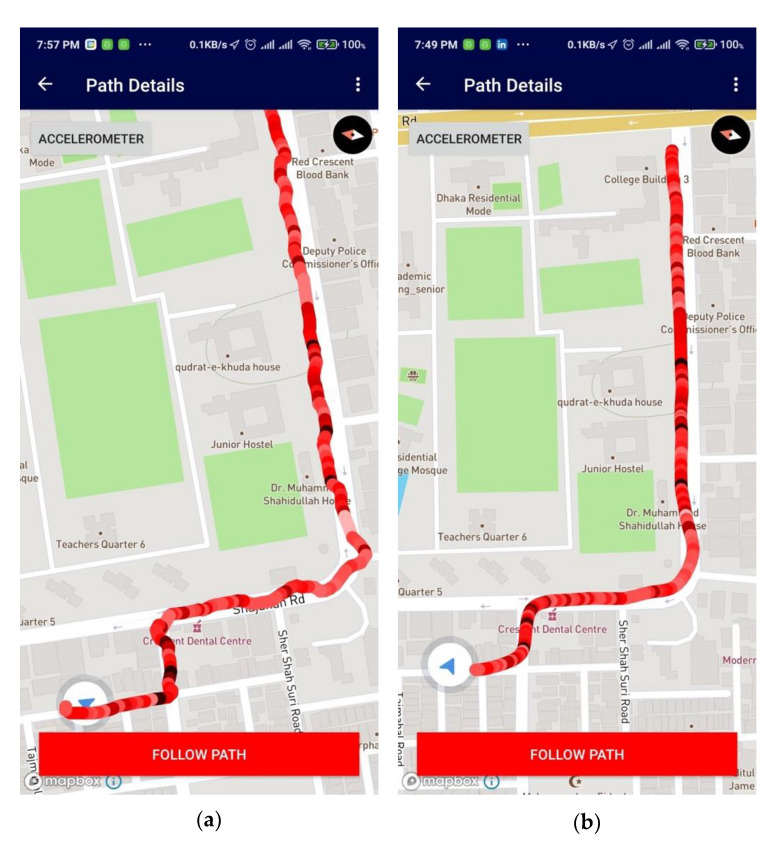
Route map created without using any filter (**a**) and after using the Kalman filter (**b**).

Details of this implementation can be found in [36]. Figure 7a shows that the routing path created in the app offers some deviations; therefore, the position vectors are placed haphazardly. The exact routing path can be fine-tuned into a smooth route after the Kalman filter’s estimation and filtering, as shown in Figure 7b. An accuracy graph on the estimate has also been provided in the Supplementary File, which defines raw position data versus filtered position data by the Kalman filter. The Kalman filter model thus increases the accuracy of position data points created by the android routing system.

### 4.4. Display of Color-Coded Intensity

**Figure 8:**
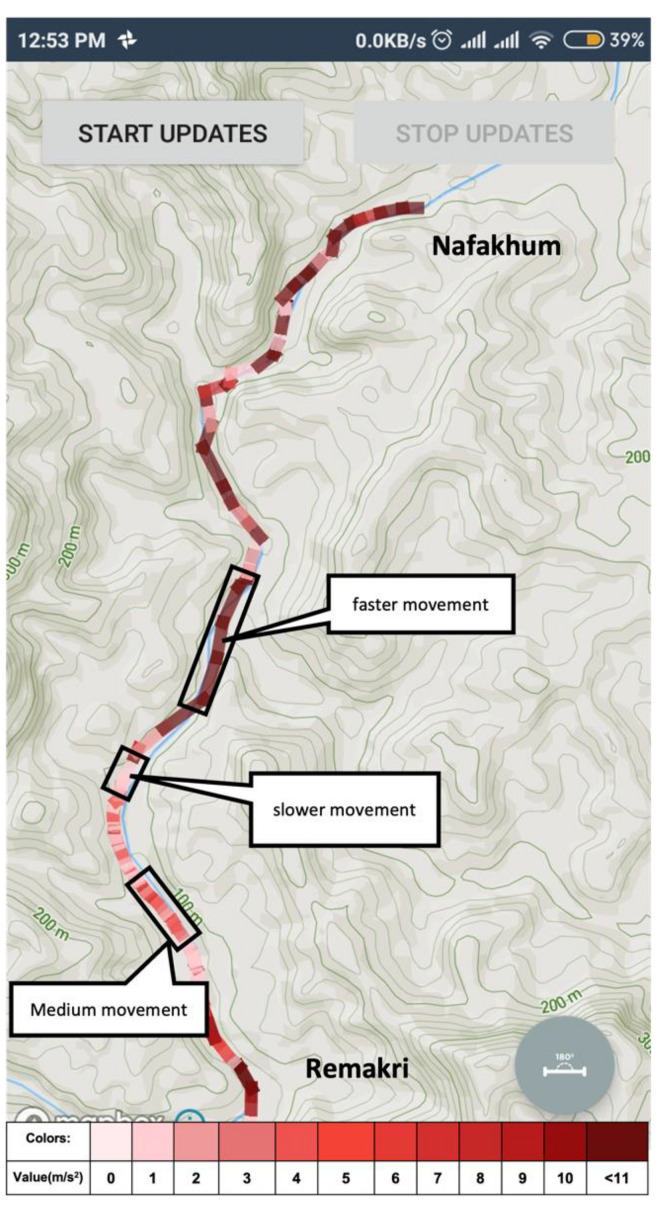
This shows that a user created a path of an unknown area from Remakri to Nafakhum falls. A new tracker can follow the route and get a clear idea of the route path through color intensity. A newbie tracker will not be lost on that hill tracks area because this map will work offline also.

When a user creates a route path, the system not only adds the GPS coordinates for the path but also stores some accelerometer data for creating a data view of the condition of that environment so that if another user wants to follow the same path, he or she can easily understand the situation of that path. The system uses the device’s accelerometer sensor for the extraction of acceleration values. A sensor of this type measures the acceleration applied to the device (Ad). Conceptually, it does so by measuring forces applied to the sensor itself (Fs) using the relation: Ad = −∑Fs/mass. Smartphones gives the gravity value with acceleration data (gravitational acceleration-g). However, gravity constantly influences the measured acceleration and mass value is constant, which is received from a smartphone [37] and, hence, Ad becomes [−g − ∑Fs/mass]. The sensor values are sent to the database to store with the respective map. The use of gravitational acceleration to deduce altitude with color coded intensity is presented in the mapping application as shown below in Figure 8. 

### 4.5. Battery Consumption

Smartphones are embedded with sensors from various vendors. As a result, the power consumption level differs from device to device. Table 3 provides a battery consumption comparison among different Smartphone models of the same brand and other brands while running the required sensors. The maximum total discharge rate recorded is about 4% and average minimum total discharge rate is about 1.3% for efficient devices, as can be seen in the table below. However, it can also be seen that a device (Oppo F11 Pro) was found to have an extremely lower discharge rate, which was about 0.004%. This can be due to a bug within the OS, which shows the sensor of this device is consuming much less current than actual or that the device is consuming very low power and, thus, highly efficient. Due to a lack of manufacturer information, it is not possible to give any definitive information regarding this. Fortunately, the Android Sensors API provides a method for extracting each sensor’s power consumption level in Milli Amperes (mA). Testing the application was done in five different devices, and the four sensors’ power was measured. The battery percentage consumed by each sensor was calculated by comparing it with each device’s battery capacity for a more precise representation (prices are based on the latest information available on Amazon).

Power consumed while fetching a location is quite challenging to track. Google Location Services API, mainly the fused location API, is implemented to achieve this. Location gathering and battery drain are directly related to accuracy, frequency, and latency. The API uses GPS, Wi-Fi, cell services, and various sensor data for high precision, which can cause significant battery drain. The frequency and latency can be set programmatically; lowering the interval may result in higher battery consumption. The proposed application requires a frequency and latency of 9000 milliseconds to ensure precision, which puts a relatively low strain on the battery considering its features. For high frequency, it required huge data storage and more Random-Access Memory (RAM). Additionally, the Operating system (OS) kills the sensor data access service.

## 5. Experiments and Results

The users travelled numerous areas with the proposed application. The collected data were processed, and the results were evaluated to check if the outcome was better than what is currently available. The locations were mainly compared with Google Maps as it is vastly used worldwide and is very popular.

### 5.1. User Authorization and Credential

**Figure 9:**
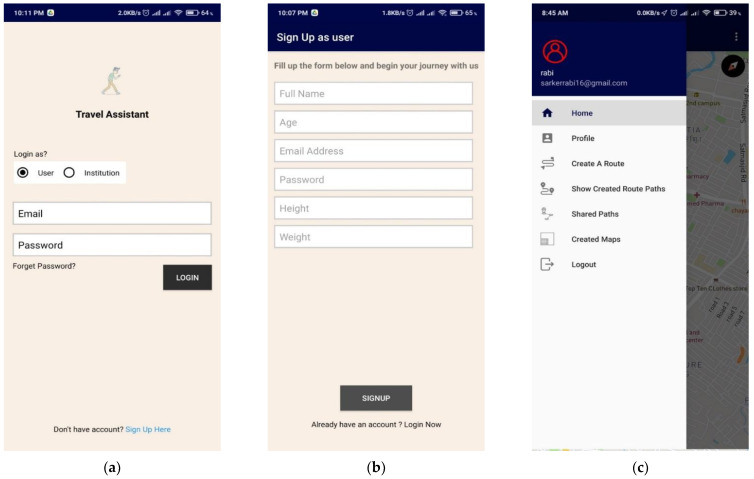
Application layout of Login (**a**), Sign up (**b**), and Profile (**c**).

To use the application, an individual or an institution needs to sign up first, as shown in Figure 9. Both types of users are verified using their personal/institutional email address. The term ‘institution’ used here refers to the administrative body of public places such as shopping malls, universities, stores, parking areas, and restaurants. The individual can sign up as a regular user using a personal email address or an institution’s authority using an institutional email address. In the sign-up process, the user needs to input some basic info such as weight, height, and age, and the user can get access to the application immediately. In order to sign up as an institution that can create or validate the surface map, one needs to provide an exact address and select the surface’s location from the map. The application authority (admin) of the application needs to verify their data before logging in and creating a verified map for their place. All user data are saved into the firebase real-time database.

### 5.2. Path Tracking

**Figure 10:**
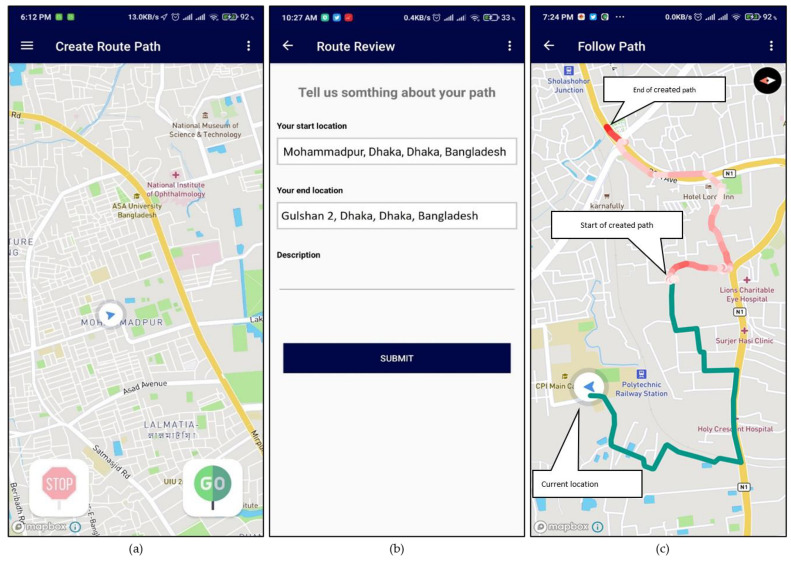
The initial view of (**a**) create a route path, (**b**) after making a path, and (**c**) follow a path option of the mobile application.

The application can track the user path in real-time as the user moves, as shown in Figure 10a below. Using these features, users can create maps of any location that they visit. The path is built primarily using GPS coordinate readings fetched by the application. Furthermore, the pathways are shown in a map with different color coding based on sensor readings during the journey. The color-coded representations are beneficial for rough mapping terrains and vertical movement. Figure 10b shows that the user can add details of their experiences of the new route with start and end location info. Hence, it is a preview option to get better feedback on crowdsourcing and understanding of that route. Figure 10c shows three points in that area: current user location, starter location, and end location of the previously created path. If the user’s current location is far from the start location, the system automatically generates a path for the user to follow shown in green. The previously created path is leading with the sensor-based color generation. In order to access paths recorded by other users, one has to select the option called Shared Paths (Supplementary Material Figure S1a). There, he or she will find the recorded paths (Supplementary Material Figure S1b) shared by other users. Then, selecting their desired paths, they can see the shared path (Supplementary Material Figure S1c) and follow the created path (Supplementary Material Figure S1d).

Multiple mapped routes may be available for a specific source-destination since any user traveling through the same path has the option to create and share it. If multiple maps are available for the exact locations, users may select the map based on other users’ reviews. Pre-loaded maps can serve the travelers and be convenient for the delivery people who regularly provide service in a particular area.

### 5.3. Map Creation for the Unreached Area

**Figure 11:**
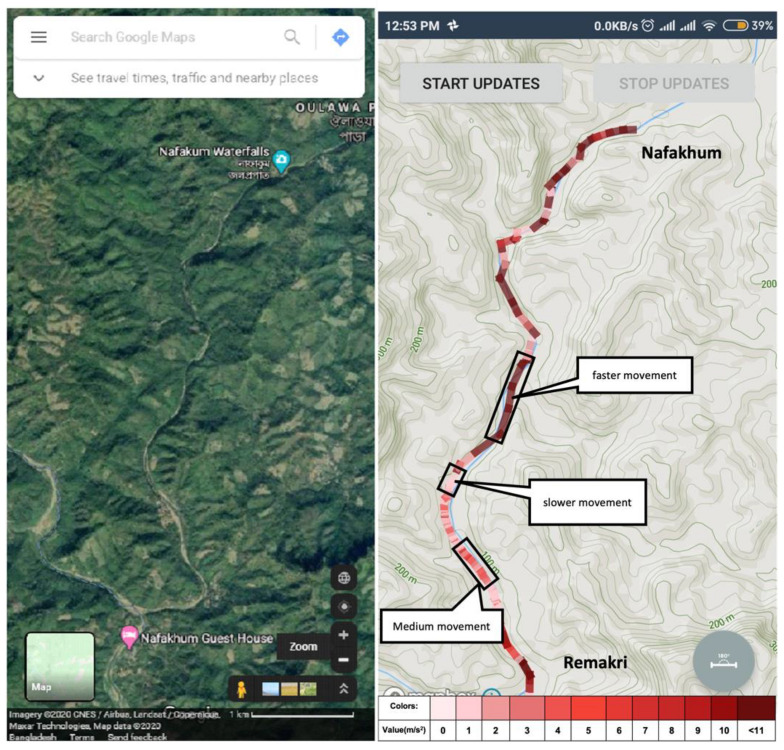
Top view from Google Maps (**Left**) and the top view from the proposed app (**Right**).

For navigating remote areas, adventurous travelers also need a guide for touring that place, especially when popular maps cannot help users find a path to go to that place. Using this system, if a user creates a route for an unknown location, he or she establishes the system’s route path and paves the way for others to travel to that place. Nowadays, bloggers can easily create a video blog for an adventure place, and with such resources, a traveler can get a visual of the location. However, if someone likes to track the same place, video resources might not be helpful. With the proposed application, when the user presses the start button to create a route path, the system saves the coordinate of that location. It collects other valuable sensor data for keeping the condition of that point. In Figure 11, an unknown area situated at Bandarban districts, Chittagong, Bangladesh, is shown. A user creates a route path for travelling to a place named Nafakhum falls from Remakri falls. Google Map, or other popular maps, shows only the name of those places, but it does not show any direction.

### 5.4. Surface Map Creation

Surface maps of any public locations such as shopping malls, academic buildings of a University, and other mega infrastructure or industries can be produced and shared using the proposed system. One of the most widely used mapping applications, Google Maps, already provides such a feature. However, Google Maps’ indoor map feature covers only about 60 countries [38], primarily well-developed countries. On the contrary, the proposed system would work in every country and have better coverage as it mainly relies on crowdsourcing.

**Figure 12:**
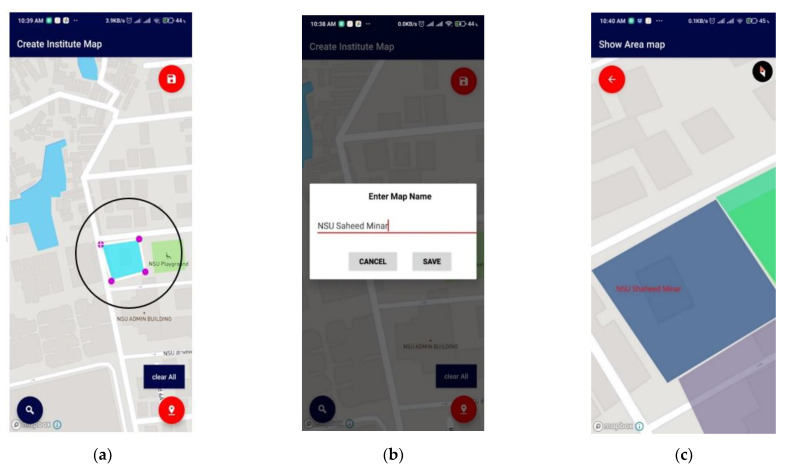
Surface map creation of an Institution (**a**) by selecting an area, (**b**) setting a name of the chosen location, and (**c**) displaying created map, which is visible to all users.

Institutions registered with the application hold all rights to create and edit their surface map. The map can be created manually by drawing over a map layout with the application and physically moving along the structure, keeping the application tracker on. A map can be made using *create a surface map* option. When clicked onto this option, the system provides a point/marker on the 2D plane. The user can add the points and map their authorized areas, as shown in Figure 12.

**Figure 13:**
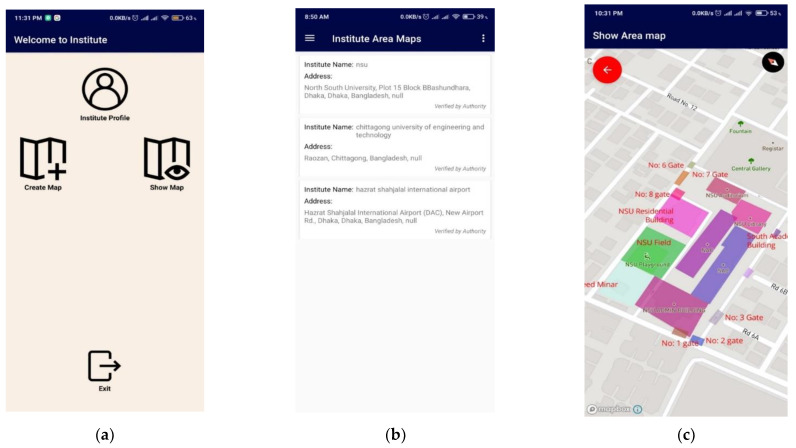
Applications’ layout of (**a**) Institutional user window, (**b**) list of saved Institutional maps, and (**c**) showing the saved Institutional map.

Surface maps of every registered institution are stored in the cloud and are accessible to all users. Individual users can search for particular places by their addresses using the application, and the corresponding surface maps are presented. It will create a new layer on the existing map with the cloud data and background map data. Figure 13 shows other searchable addresses for an institution during sign-up to help users find the place easily.

### 5.5. Surface Map of Public Places

**Figure 14:**
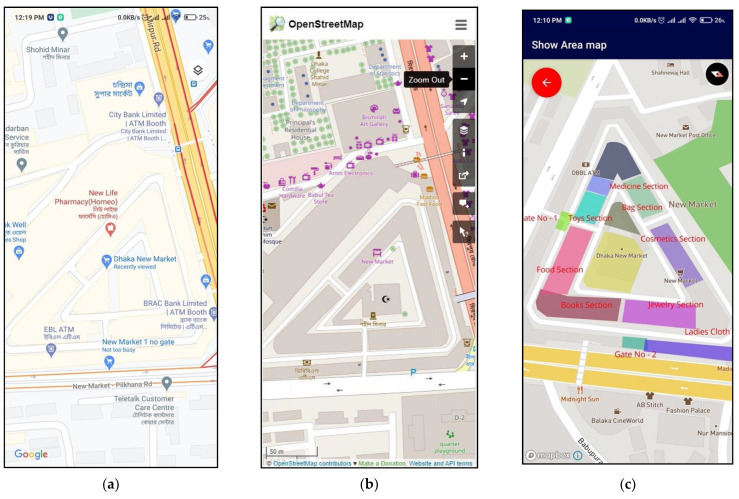
(**a**) Google Map view, (**b**) OpenStreetMap view, and (**c**) proposed application.

For the experiment, two famous places were considered in Bangladesh. Dhaka New Market was chosen as a sample location for area mapping. As shown in Figure 14a, the Google Maps’ application view is less informative. Section-wise, area locations are not included in the representation. In Figure 14b, The OpenStreetMap view shows only the market name and building area, nothing more. Moreover, details can be found in Figure 14c from an authorized user using the proposed application.

**Figure 15:**
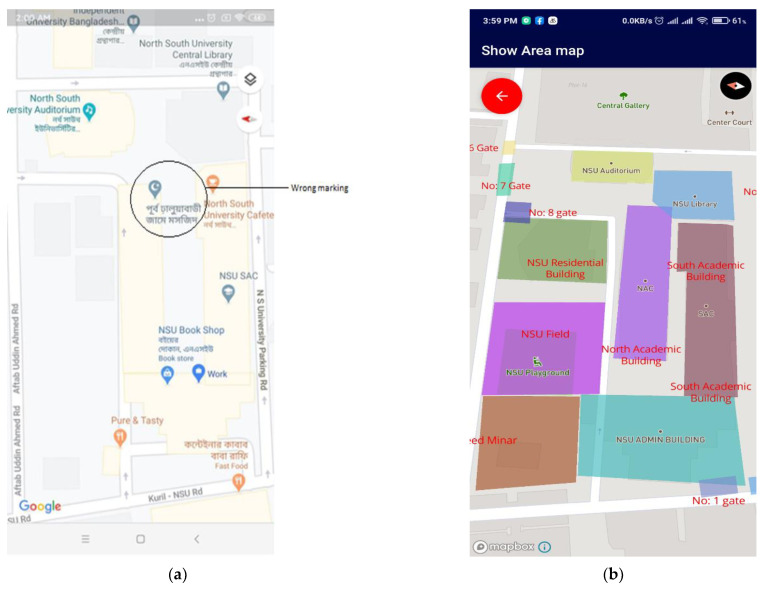
Top view from Google Maps (**a**) and the top view from the proposed application (**b**).

For creating a surface map of an academic building, North South University, situated in Bashundhara Residential Area, Dhaka, Bangladesh, was chosen. The institutional map by Google Maps and the proposed application are given in Figure 15a,b, respectively. The upper view is shown by both applications, which does not differ much. Figure 15b contains details needed by a student to explore the area, which are missing in Figure 15a.

## 6. Discussion 

In this work, it was hypothesized that the smartphone sensors can be used to create route maps of unreached areas and surface maps of infrastructure efficiently. The number of route maps can also be increased by using crowdsourcing. The travelers’ exploration can be made useful to others. In the context of the hypothesis, a software architecture was proposed and implemented and the tests were carried out to show that the expectations based on the hypothesis can be fulfilled. The Fused locator API from Google Play store was used to retrieve the GPS information from the sensors (Figure 6), and the required list of sensors and software was provided in Table 1. The filtration technique to improve the quality of GPS information was used (Figure 7). The GUI with easy-to-use, simple steps (Figure 10) and features for creating and sharing tab for routing and mapping options should attract crowdsourcing. The hypotheses were tested by obtaining the accuracy of the mapping application (Figure 14 and Figure 15) and by the creation and sharing of route paths (Figure 7 and Figure 8) and surface maps (Figure 13 and Figure 14). The source code and proposed application can be found in the given references [39,40].

A few other existing mapping applications were investigated, and detailed comparisons were made (in Table 4). It is evident from the table that almost none of the mentioned applications have the feature called *Surface mapping*. The proposed app has almost all the common features a mapping app should have for personal usage and a unique feature called surface mapping that offers an advantage to the users to see area mapping of famous places, markets, or universities with accurate information verified by the respective authorities. Moreover, the app’s design is simple as it does not require the hassle of uploading GPX files, KML files, or any images. Thus, creating routes, mapping the unmapped regions, sharing the routes with other users, or making routes for personal use make the proposed app user friendly and valuable at the same time. In the comment column of the table, some key observations were noted while surveying the applications. 

## 7. Conclusions

The proposed solution creates a crowdsourcing platform where human resources generate verified area and route paths on the maps for building a robust navigation system. It can also help users to find desirable places in mega infrastructures easily provided by the authority. It is an excellent platform for adventure-minded people to create a path of newly explored areas and share it with others so that anyone can explore these places easily. People can also personalize their frequently visited destinations’ paths to track them easily. User participation in map production is vital for growing the coverage in the shortest possible time. However, it may create issues with the reliability of the data. Existing popular map solutions do not provide any systematic quality check of the data. In the proposed solution, it has been partially solved by keeping a verification process for the respective authority’s surface map creation. 

In the future, a 3D view will be created using a color template in the GUI to enhance the user experience. With this, users can get information of variance in the height of the terrain they wish to go. The calorimeter template is still under development and will be provided for the application in later stages. Therefore, the user may also receive different readings such as step count, time duration, and total distance travelled after the application is turned on, and heartbeat during the travel.

## Figures and Tables

**Table 1:** Android Sensors API version support for sensors.

Sensors	Android 4.0 (API Level 14)	Android 2.3 (API Level 9)	Android 2.2 (API Level 8)	Android 1.5 (API Level 3)
Accelerometer	Yes	Yes	Yes	Yes
Gyroscope	Yes	Yes	deprecated	deprecated
GPS	Yes	Yes	Yes	n/a
Magnetometer	Yes	Yes	Yes	Yes

**Table 2:** Minimum data retrieval rate required per sensor.

Sensor	Minimum Delay
Acceleration Sensor	0 or on data changes
Gyroscope	100 ms
Magnetometer	0 or on data changes
GPS	n/a

**Table 3:** A comparison of battery consumptions of the sensors on various devices.

Device Name	Android OS	Battery Capacity (mAh)	Price (USD)	Battery Consumption			Total Battery Consumption (mA)	Total Percentage Charge (Per 24 h)
				Acceleration Sensor (mA)	Gyroscope (mA)	Magnetometer (mA)		
Xiaomi Redmi 4x	7.1.2	4100	110	0.4	2.3	1.0	6.525	3.82%
Xiaomi Redmi Note 6 Pro	8.1	4000	190	0.325	2.5	1.1	6.75	4.05%
Xiaomi Redmi Note 7	9.0	4000	250	0.15	0.555	1.1	2.32	1.39%
Oppo F11 Pro	9.0	4000	289	0.001	0.001	0.001	0.01	0.004%
OnePlus 7 Pro	9.0	4000	629	0.15	0.555	1.0	2.22	1.33%
Google Pixel 2 XL	8.0	3520	950	0.15	0.45	1.8	3.40	2.32%
Samsung Galaxy Note 10+	9.0	4300	950	0.17	0.55	1.1	2.34	1.30%

**Table 4:** Comparison between some similar mapping apps and the proposed mapping app.

Mapping Apps	Proposed Features		Common Features				Price	Service Status	Comments
	Route Mapping	Verified Surface\Area Mapping	Display Map	Navigation	Make Track	Monitor			
Osmdroid	Yes	No	--	no	no	no	Free	Unmaintained	Replaces GMaps’ views, OSM based, Not updated over a year
OSM-Tracker for Android	--	No	yes	no	yes	yes	Free	Active	Application is not updated with latest devices
Trekarta	Yes	No	Yes	yes	yes	yes	$0.99, free	Active	Designed for outdoor activities only
OsmAnd	Yes	No	Yes	yes	yes	yes	Free with limited options; $11.8	Active	Free Version is too slow to render; lacks refinement with address searching; limited searchable address;
Routes- GPX KML Navigation & GPS Tracker	No	No	yes	yes	yes	yes	Free	Active	Designed for outdoor activities; requires GPX/KML file to upload
Vespucci	--	No	yes	no	yes	--	Free	Active	OSM Based requires an OSM account, needs maintenance
Footpath Route Planner	--	No	yes	yes	yes	--	~$2/month		Active Suitable for outdoor activities, running/hiking only
Komoot	--	No	yes	yes	yes	yes	Free	Inactive	It requires more than usual time to operate
AndRoad	--	No	yes	yes	yes	No	Free	Inactive	Inactive since 2011
Proposed Map—Travel Assistant	Yes	Yes	No	yes	yes	yes	Free	Proposed	

## Supplementary Materials

The following are available online at https://www.mdpi.com/article/10.3390/s21216969/s1, Figure S1: one has to select the option called Shared Paths (a). There, he or she will find the recorded paths (b) shared by other users. Then, selecting their desired paths, they can see the shared path (c) and follow the created path (d). Figure S2: Raw position data versus filtered position data by Kalman filter. It can be seen blue dots represent position vectors without use of filter whereas the orange ones rectify by estimating difference in position vectors and creates a much smoother and precise data points. Figure S3: Database schema of the proposed application. Figure S4: Sign up view of Institutional user. Figure S5: Sign up view of traveller. Figure S6: Navigation view of traveller. Figure S7: Route history of created path. Figure S8: List of Surface maps. Figure S9: List of Surface maps. Figure S10: Other user’s shared path list. Figure S11: Another created path in a remote area, Video S1: Create Surface map. Video S2: Create route path share with others. Video S3: Search Shared Surface map.
