# Supplementary material for: An Efficient Surface Map Creation and Tracking Using Smartphone Sensors and Crowdsourcing
Source: Sensors (Basel). 2021 Oct 20;21(21):6969. doi: 10.3390/s21216969 (PMC8587749; doi:10.3390/s21216969)
Supplement: Supplementary file 1 [file sensors-21-06969-s001.zip › Supplementary Materials.pdf]

Supplementary Materials

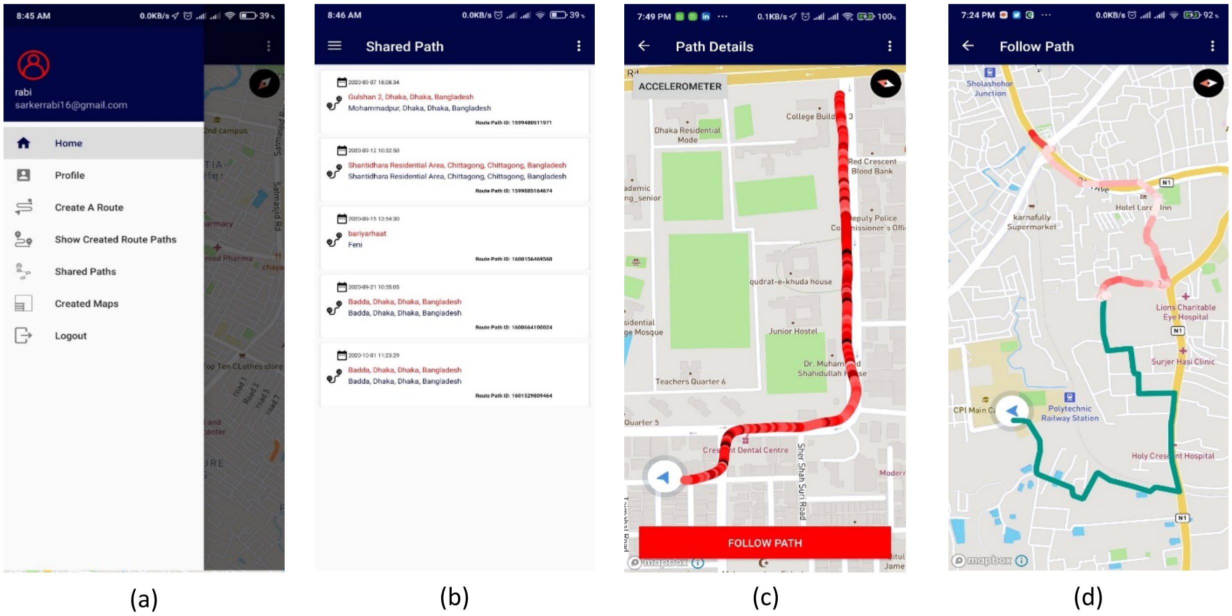

Figure S1. One has to select the option called Shared Paths (**a**). There, he or she will find the recorded paths (**b**) shared by other users. Then, selecting their desired paths, they can see the shared path (**c**) and follow the created path (**d**).

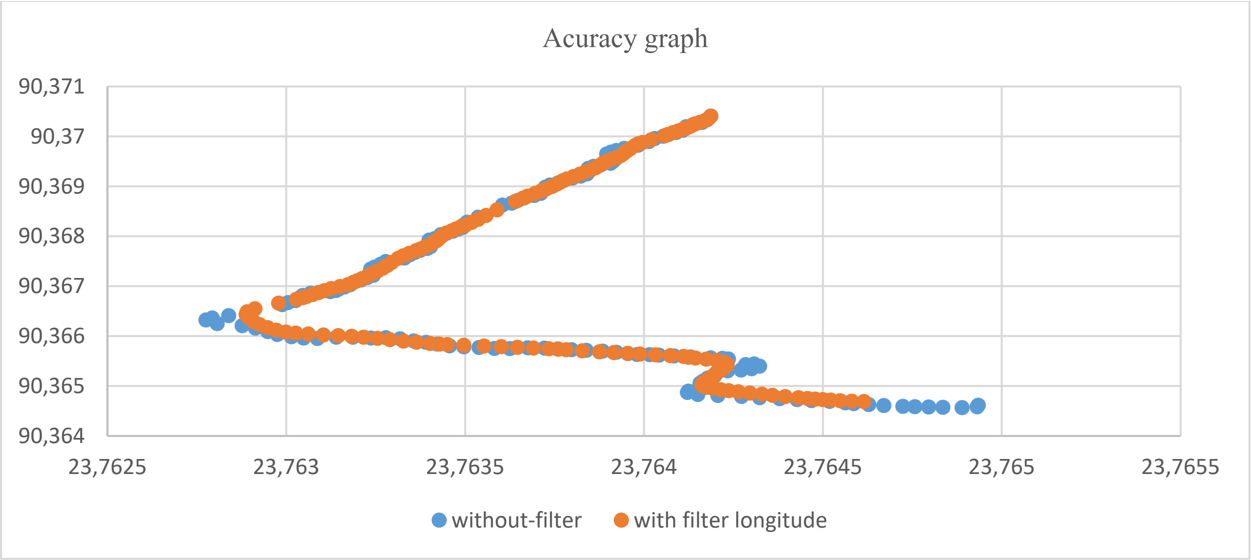

Figure S2: Raw position data versus filtered position data by Kalman filter. It can be seen blue dots represent position vectors without use of filter whereas the orange ones rectify by estimating difference in position vectors and creates a much smoother and precise data points.

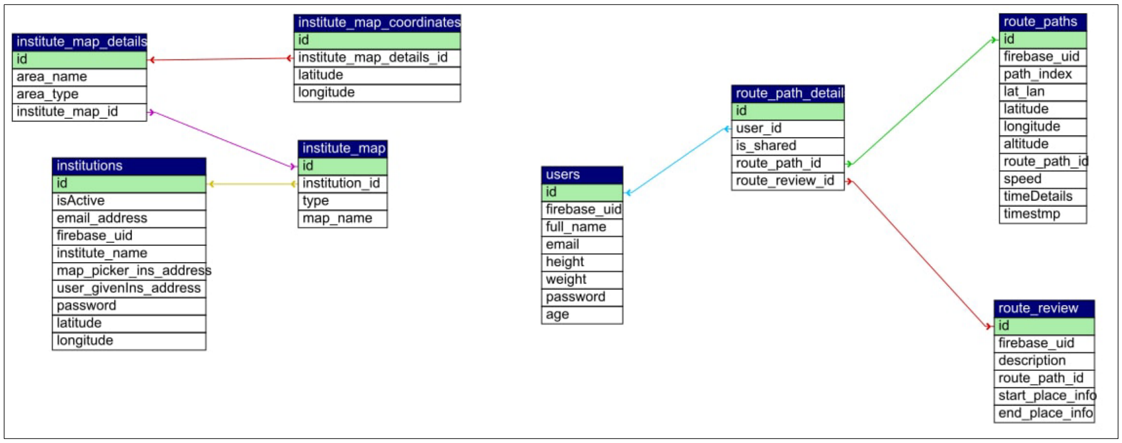

Figure S3: Database schema of the proposed application

Some other figures from proposed application

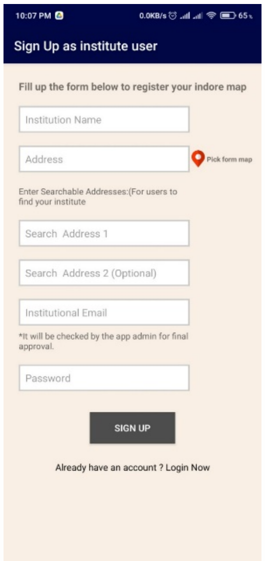

Figure S4: Sign up view of Institutional user

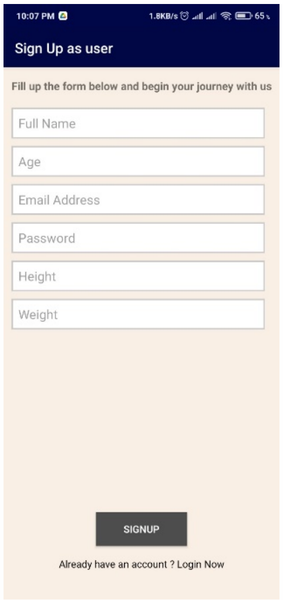

Figure S5: Sign up view of traveller

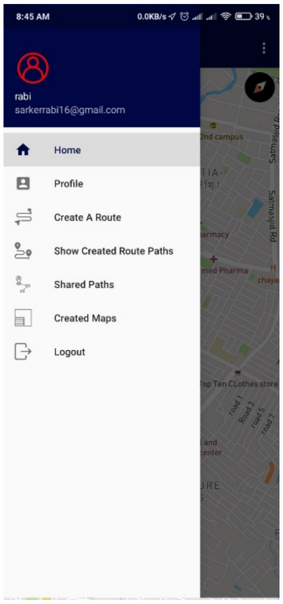

Figure S6: Navigation view of traveller

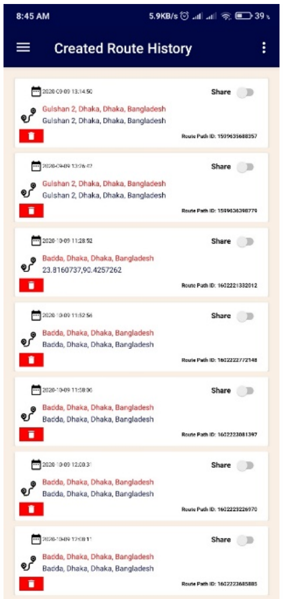

Figure S7: Route history of created path

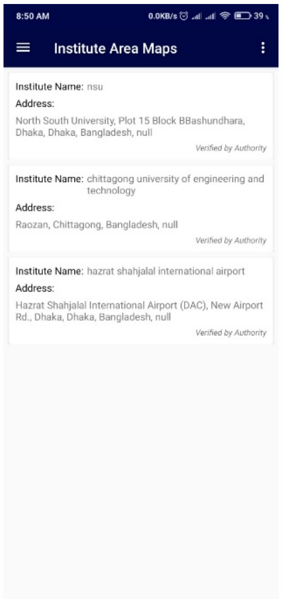

Figure S8: List of Surface maps

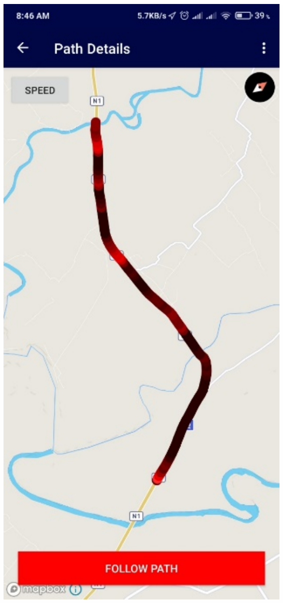

Figure S9: Another created path view

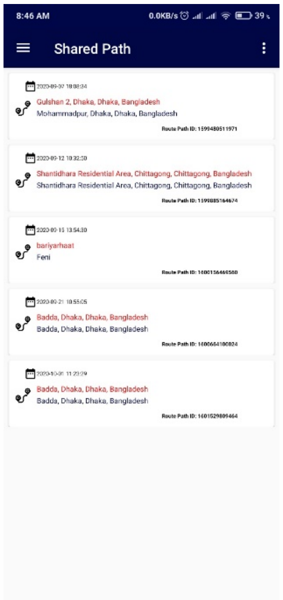

Figure S10: Other user ‘s shared path list

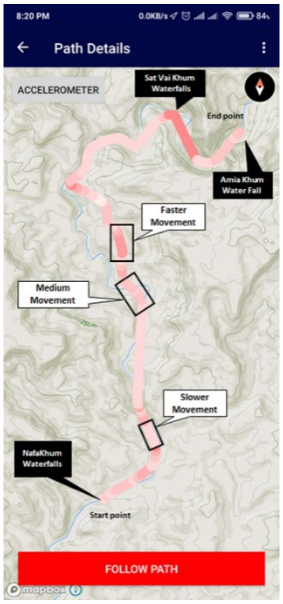

Figure S11: Another created path in a remote area
